# Psychotropic medication non-adherence and its associated factors among patients with major psychiatric disorders: a systematic review and meta-analysis

**DOI:** 10.1186/s13643-020-1274-3

**Published:** 2020-01-16

**Authors:** Agumasie Semahegn, Kwasi Torpey, Adom Manu, Nega Assefa, Gezahegn Tesfaye, Augustine Ankomah

**Affiliations:** 1 Department of Population, Family and Reproductive Health, School of Public Health, College of Health Sciences, University of Ghana, Accra, Ghana; 2 College of Health and Medical Sciences, Haramaya University, Po Box 235, Harar, Ethiopia; 3 Population Council/Ghana, Yiyiwa Drive, Accra, Ghana

**Keywords:** Medication non-adherence, Psychiatric disorders, Systematic review, Meta-analysis

## Abstract

**Background:**

Major psychiatric disorders are growing public health concern that attributed 14% of the global burden of diseases. The management of major psychiatric disorders is challenging mainly due to medication non-adherence. However, there is a paucity of summarized evidence on the prevalence of psychotropic medication non-adherence and associated factors. Therefore, we aimed to summarize existing primary studies’ finding to determine the pooled prevalence and factors associated with psychotropic medication non-adherence.

**Methods:**

A total of 4504 studies written in English until December 31, 2017, were searched from the main databases (*n* = 3125) (PubMed (MEDLINE), Embase, CINAHL, PsycINFO, and Web of Science) and other relevant sources (mainly from Google Scholar, *n* = 1379). Study selection, screening, and data extraction were carried out independently by two authors. Observational studies that had been conducted among adult patients (18 years and older) with major psychiatric disorders were eligible for the selection process. Critical appraisal of the included studies was carried out using the Newcastle Ottawa Scale. Systematic synthesis of the studies was carried out to summarize factors associated with psychotropic medication non-adherence. Meta-analysis was carried using Stata 14. Random effects model was used to compute the pooled prevalence, and sub-group analysis at 95% confidence interval.

**Results:**

Forty-six studies were included in the systematic review. Of these, 35 studies (schizophrenia (*n* = 9), depressive (*n* = 16), and bipolar (*n* = 10) disorders) were included in the meta-analysis. Overall, 49% of major psychiatric disorder patients were non-adherent to their psychotropic medication. Of these, psychotropic medication non-adherence for schizophrenia, major depressive disorders, and bipolar disorders were 56%, 50%, and 44%, respectively. Individual patient’s behaviors, lack of social support, clinical or treatment and illness-related, and health system factors influenced psychotropic medication non-adherence.

**Conclusion:**

Psychotropic medication non-adherence was high. It was influenced by various factors operating at different levels. Therefore, comprehensive intervention strategies should be designed to address factors associated with psychotropic medication non-adherence.

**Systematic review registration:**

PROSPERO CRD42017067436

## Background

Psychiatric disorders have been a global public health challenge. Almost 450 million people are affected by psychiatric disorders worldwide. It contributes 14% of the overall global burden of diseases, and 30% of the non-fatal diseases burden, which is worsened by medication non-adherence [1–3]. Psychiatric disorders cost approximately US$2.5 trillion in 2010 and are expected to rise up to US$6.0 trillion by 2030. Lost resources and production, unemployment, absences from work, and premature mortality are some of the indirect economic costs [3]. The World Health Organization (WHO) has designed a comprehensive strategic action plan (2013–2020) to promote mental well-being, prevent psychiatric disorders, and provide care and support to reduce morbidity, disability, and mortality [4].

Nearly one third (31.7%) of people who suffer major psychiatric disorders end up with a long-term disability and dependency [5]. Psychiatric disorders are associated with individual factors as well as community social support, cultural, social protection, living standards, and other environmental factors [4]. Compliance to medication is essential but challenging in the management of major psychiatric disorders [6–8]. The WHO defines medication non-adherence as, “a case in which a person’s behavior in taking medication does not correspond with agreed recommendations from health personnel” [9]. Patients with major psychiatric disorders are most likely to be non-adherent to medication due to poor reasoning and lack of insight about their illness and treatment [8, 10, 11].

Psychotropic medication non-adherence can lead to exacerbation of their illness, reduce treatment effectiveness, or leave them less responsive to subsequent treatment. Other consequences of non-adherence include re-hospitalization, poor quality of life or psycho-social outcomes, relapse of symptoms, increased co-morbid medical conditions, wastage of health care resources, and increased suicide [7, 8, 12–15]. Research evidence on the level of psychotropic medication non-adherence and its associated factors among patients with major psychiatric disorders is essential to design appropriate interventions to achieve desired treatment goals for both patients and health care providers. Although several primary studies have been conducted on this issue, there has not been any systematic review and meta-analysis carried out to inform policy. Thus, a systematic review and meta-analysis on the level and factors associated with psychotropic medication non-adherence is useful to inform policy makers and program planners. Therefore, the main aim of this systematic review and meta-analysis was to summarize available findings of primary studies to determine the level of psychotropic medication non-adherence and associated factors.

## Methods

### Protocol development and registration

This systematic review and meta-analysis has been registered in the international Prospective Register of Systematic Reviews (PROSPERO 2017:ID:CRD42017067436) [16] and written in accordance with the Preferred Reporting Items for Systematic Review and Meta-analysis (PRISMA) statements guidelines [17] (see Additional file 1). The detail of this systematic review and meta-analysis protocol has been published elsewhere [18].

### Search methods for identification of studies

The Medical Subject Headings (MeSH) and keywords were constructed based on the review question. Studies were searched using search engines, from the main electronic databases (PubMed (Medline), EMBASE, CINAHL, Web of Science, and PsycINFO), and other sources (Google Scholar, reports, thesis, or dissertation). Search strings were constructed using a combination of MeSH terms such as psychotropic non-adherence, non-compliance, compliance, adherence, determinants, barriers, associated factors, risks, correlates, influencing factors, and major psychiatric disorders (see Additional file 2). The search strings were modified to suit to the corresponding database interface. All of the identified studies were exported to the EndNote citation manager [19], and duplicates were removed.

### Eligibility criteria

Studies were included in the systematic review and or meta-analysis if they fulfill the following eligibility criteria. The criteria were as follows:

- Studies had been conducted among adult patients (18 years and older);
- Studies had been conducted on one or more of the major psychiatric disorders (major depressive disorders, schizophrenia, or bipolar disorder) were eligible;
- Studies reported psychotropic medication non-adherence or adherence and or factors associated with medication non-adherence;
- Studies conducted at community and/or facility-based;
- Studies used observational study designs (cross-sectional, case-control cohort, and or survey);
- Studies were written in English before December 31, 2017;
- Documents (both published and unpublished studies, survey reports, thesis, or dissertations) which were accessible with full text.

### Selection of studies into systematic review

**Fig. 1:**
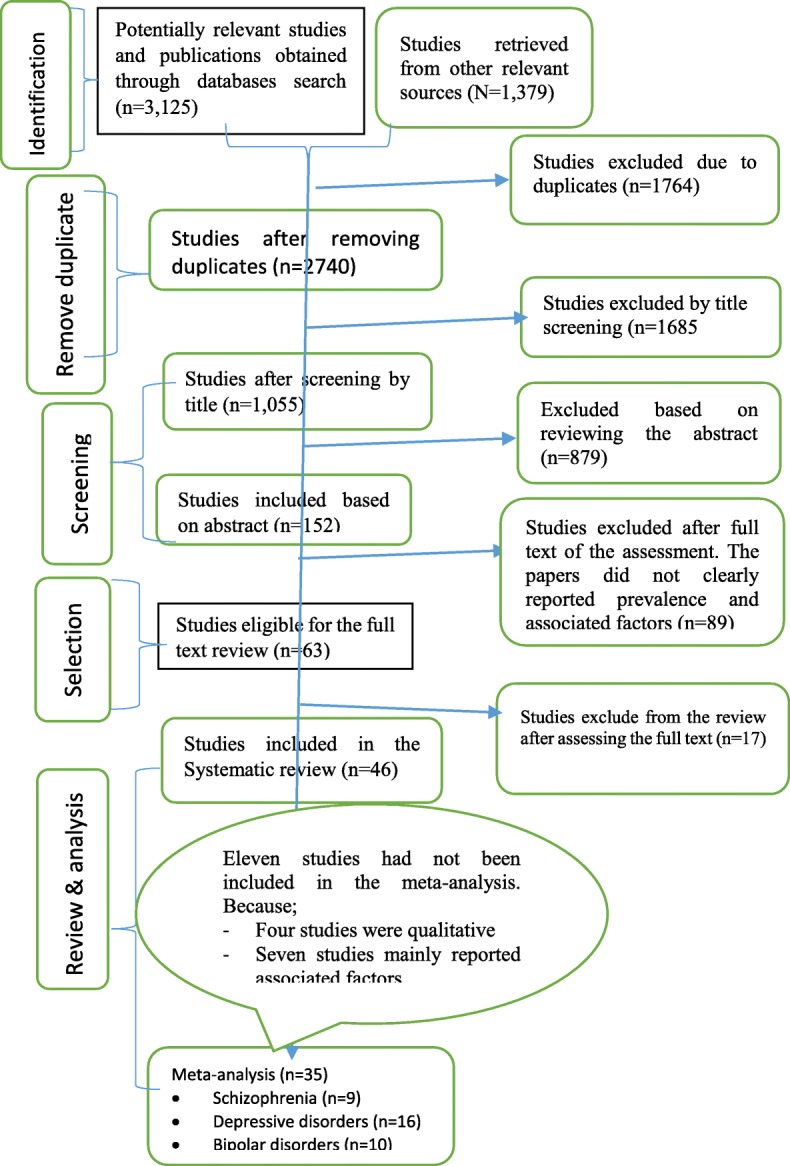
Diagrammatic presentation of the selection process of studies for systematic review and meta-analysis

Studies were systematically selected using predetermined eligibility criteria. Studies’ title and abstract that clearly mentioned either patients with major psychiatric disorder psychotropic medication non-adherence or adherence were selected for the subsequent evaluation. Then, to minimize bias during screening, two authors (AS and GT) independently screened the title and abstract of the studies to proceed to the next step of the studies selection. Studies overview such as aim of the study, design of the study, participants, and main outcome of the study were screened. In this stage, the studies potentially eligible for the full text were selected based on the title and abstract. The full text of the studies selected based on the title and abstract were re-assessed independently by two of the authors (AS and GT) for details. The body of the studies’ (aims, mainly design, participants, sampling method, findings, conclusions, and recommendations) were assessed. Finally, studies have reported the medication adherence or non-adherence among major psychiatric disorders patients (schizophrenia, major depressive, or bipolar disorders) and associated factors and fulfill the eligibility criteria were selected for the systematic review and meta-analysis. All studies that consider psychiatric disorders as a factor for medication non-adherence were excluded, because studies that consider psychiatric disorders as predictor for the non-adherence to treatment for other illness may not fully assess the adherence level of psychotropic medications. Overall, the studies’ selection process was adhered to the PRISMA flow diagram [17] (Fig. 1). Any difference during studies selection process was resolved through consensus.

### Measurement of outcome and exposure

According to the WHO, medication non-adherence is defined as “a case in which a person’s behavior in taking medication does not correspond with agreed recommendations from a health personnel”. It can be either intentional or unintentional, including failing to initially fill or refill a prescription, discontinuing a medication before completing the course of therapy, taking more or less of a medication than prescribed, and taking a dose at the wrong time [9]. Thus, the main outcome of interest for this systematic review was the level of psychotropic medication non-adherence. Medication non-adherence was measured either as direct report from studies or indirectly by subtracting adherence report from total observations (sample size). Studies’ reported non-adherence in another way such as medication non-compliance, non-persistence, dropout, discontinuation, missing, and other alternatives was considered. Moreover, exposure or explanatory variables for the medication non-adherence were measured using synonymous terms such as determinants, predictors, barriers, associated factors, risk factors, and influencing factors.

### Quality assurance of the systematic review

We searched both published and non-peer reviewed studies comprehensively for the systematic review and meta-analysis to minimize publication bias. The electronic or computerized, manual, and email searching methods were applied to have comprehensive search. Eligibility criteria, selection method, quality assessment, data extraction template, and regular meeting for discussion schedule were pre-designed by authors to assure the quality. The studies’ methodological quality critical appraisal was carried out using the Newcastle-Ottawa Scale [20] (see Additional file 3).

### Data abstraction, synthesis, and statistical analysis

The two authors (AS and GT) abstracted the data from the included studies and recorded in the data extraction template. Studies’ detail descriptions such as an author, study area or country, aim, design, sample size, sampling procedure, and response rate were presented on the table using Microsoft Word (2013) (Table 1). Meanwhile, the raw data of medication non-adherence and total sample size were extracted and stored using Microsoft Excel (2013) template (see Additional file 4). All the meta-analysis were carried out using Stata SE-64 version 14.2 (Stata Corporation, College Station, TX) [66] and based on the recommendation for the meta-analysis of observational studies [67]. Heterogeneity between studies was assessed and substantial heterogeneity was anticipated when *I*^2^ greater than 75% [68, 69]. The pooled prevalence (proportion) was estimated using the inverse variance method [66]. The 95% confidence interval for pooled and sub-group proportion of patients’ medication non-adherence was computed. Moreover, the sub-group-pooled proportion of patients’ medication non-adherence was performed for schizophrenia, major depressive disorder, and bipolar disorders separately. Random effects model [70] was used for the overall pooled estimate and sub-group meta-analysis.

**Table 1:** Description of studies included for systematic review and meta-analysis (*n* = 46)

Author, country	Study aim	Design	Population	Sampling methods	Sample size	Scale used	Response rate (%)
Ibrahim et al., Nigeria [21]	To determine the socio-demographic and clinical predictors of sub-optimal MA	CS	Schizophrenia & depression patients	SRS	390	MMAS	94.8
Alene et al., Ethiopia [22]	To evaluate MA and associated factors	CS	Schizophrenia patients	Purposive	336	CFR	87.5
Eticha et al., Ethiopia [23]	To investigate factors associated with MA among patients with schizophrenia	CS	Schizophrenia patients	Consecutive	393	MARS	97.5
Kenfe et al., Ethiopia [24]	To assess the magnitude and associated factors of MNA	CS	Psychiatric patients	Consecutive	422	MMAS	100
Hibdye et al., Ethiopia [25]	To assess the prevalence and factors associated with MNA	CS	Bipolar disorders patients	Systematic	410	MMAS	97
Anne et al., USA [26]	To examine the barriers of antidepressant MA	Longitudinal	Depression patients	Multistage	134	Brief interview	90
Hill et al., Ireland [27]	To examined concurrent predictors of MNA	cohort	Psychosis Patients	Restrictive	171	Interview & DAI	NR
Moritz et al., Germany [28]	To investigate attitudes toward psychotic symptoms affect MNA	Cohort	Schizophrenia patients	Restrictive	113	Self-report questions	NR
Mert et al., Turkey [29]	To evaluate factors resulting in MNA	CS	Schizophrenic, depressive patients	Patients receiving treatment	203	SCID-I	NR
Novick et al., Multi-country-European [30]	To explore the relationship between insight and MA	CS	Schizophrenia and bipolar patients	SRS	903	MARS	NR
Hillary, Nigeria [31]	To evaluate the level of patients’ MNA and associated factors	CS	Psychiatric disorders patients	Convenient	200	MMAS	NR
Ibrahim et al., Nigeria [32]	To assessed the prevalence and exclusively X-rayed medication-related factors of MNA	CS	Schizophrenia and bipolar patients	Convenient	358	MMAS	94.2
Dibonaventura et al., USA [33]	To examine the relationship between these variables among community-dwelling patients with schizophrenia	CS	Adults schizophrenia patients	Convenience	876	MMAS	NR
Gurmu et al., Ethiopia [34]	To determine the statistical significance of the association of variables with adherence	CS	Patients who visited psychiatric clinic	Convenience	209	MARS	96.3
Magura et al., USA [35]	To identify predictors of MA among psychiatric patients	CS	Psychiatric disorders patients	Patients fulfilled eligibility criteria	131	MARS	NR
Kikkert et al., 4 European countries [36].	To explore factors influencing MA of schizophrenia patients	qualitative study	Schizophrenia patients	Purposive	91	Qualitative	NA
Teferra et al., Ethiopia [37]	To improve understanding of the underlying reasons for MA	Qualitative study	Schizophrenia patients & caregivers	Purposive	43	FGDs	NR
Sher et al., USA [38]	To evaluate the effects of caregivers’ causal beliefs about depression and their perceptions of stigma on MA	longitudinal study	MDD patients	Multistage	47	Link’s scale	NR
Mohamed et al., USA [39]	To examine the strength of association of measures of both insight and attitudes toward MA	CS	Chronic schizophrenia patients	Purposive	1432	DAI, pill count, ITAQ	NR
Sava, Turkey [40]	To investigate the relationship between treatment adherence and the level of MA	CS	Comprised of euthymic patients	Patients attending their follow-up	147	Self-report question	NR
Sirey, USA [41]	To examine the extent to which perceived stigma affected treatment discontinuation	CS	Psychiatric patients	Multi-stage	92	SCS	NR
Sajatovic, USA [42]	To examined MA among patients with bipolar disorder	Longitudinal	Bipolar patients	All	44,637	MPR	NR
Sajatovic, USA [43]	To examine antipsychotic MA among bipolar disorder	Longitudinal	Bipolar patients	All	73,964	MPR	NR
John, USA [44]	To investigate the factors associated with non-adherence	CS	Bipolar disorder patients	Interactive panel	469	Adapted tool	NR
Iseselo et al., Tanzania [45]	To determine the psychosocial problems of mental illness	Qualitative study	Patients families/care givers	Purposive	14	Interview and FGDs	NR
Olivares, Spain [46]	To evaluate long term treatment outcomes in routine clinical practice	Cohort	Schizophrenia patients	Prospective chart review	1622	GAF score	NR
Charlotte, Sweden [47]	To identify predictors of MNA to antidepressant treatment	CS	MDD patients	SRS	1031	CRF & TDM	NR
Adeponle et al., Nigeria [48]	To assess relationship of family engagement and MA	cohort	Psychiatric patients	Purposive	81	Case Note review	NR
Rashid, Malaysia [49]	To determine the treatment related risk factors with the default of depression treatment	CC	MDD patients	Convenient	148	Self-reported question	86
Roy, India [50]	To examine factors associated with poor drug compliance.	CS	Psychiatric patients	Consecutive	100	Checklist	NR
Omran, Iran [51]	To describe psychiatrists’ attributions on non-compliance	CS	Psychiatric patients	SRS	500	Interview using checklist	NR
Tara et al., Canada [52]	To assess levels of MNA and determinants	CS	Psychiatric patients	SRS	80	Self-report	NR
Banerjee, India [53]	To assess the correlates of MNA to unipolar patients	CS	Psychiatric with depression	Purposive	239	MMAS	97.2
Oliver, Spain [54]	To describe MA among patients with depression	CS	Psychiatric patients with Depression	SRS	212	Medical record	NR
Dave, UK [55]	To assess the patterns, incidence and predictors of therapy discontinuation	Cohort	MDD patients (2006–2008)	SRS	13,927	PHQ	91.2
Mahaye, South Africa [56]	To assess the levels of MA and its associated factors	CS	Psychiatric patients	Convenient	95	MMAS	NR
Sundell, Sweden [57]	To analyze whether socio-economic factors influence early discontinuation	CS	Depression patients	SRS	6536	MARS	NR
Akincigil, [58]	To describe patient and provider level factors associated with treatment adherence.	Cohort	Psychiatric patients	Convenient	4312	pharmacy claims	NR
Fawad, Pakistan [11]	To elucidate predictors of non-adherence	CS	Psychiatric patients	Convenient	128	Adapted question	94.8
Prukkanone et al., Thailand [59]	To quantify the adherence rate to and associated factors	Cohort	Depression patients	Convenient	1058	MPR	NR
Shigemur, Japan [60]	To identify predictors of antidepressant adherence	CS	MDD patients	Online survey	1151	Checklist	NR
Bambouer et al., USA [61].	To examined compliance and faxed alerts to physicians in 2003	Cohort	Psychiatric patients	Purposive	13,128	MMAS	NR
Demyttenaere et al., Belgium [62]	To investigate of compliance in patients with MDD	CS	Mdd	SRS	85	MEMS	NR
Mascha, [63]	To evaluate adherence to antidepressant among depressed patients	Cohort	Depression Patients	Purposive	131	MMAS	NR
Baldessarini et al., USA [64]	To sought risk factors to guide clinical prediction of non-adherence	CS	Bipolar patients	SRS	429	Self-report PRFs	NR
Nega et al., Ethiopia [65]	To assess psychotropic MNA and associated factors	CS	Psychiatric disorder patients	SRS	613	MMAS	92.9

*CC* case control, *CS* cross-sectional, *CRFs* case report forms, *FGD* focus group discussion, *GAF* Global Assessment of Functioning score, *ITAQ* Insight and Treatment Attitudes Questionnaire, *MA* medication adherence, *MNA* medication non-adherence, *MMAS* Morisky Medication Adherence Scale, *CFR* compliant fill rate, *MARS* Medication Adherence Rating Scale, *MEMS* Medication Monitoring System, *MPR* Medication Possession Ratio, *NRR* no-response rate, *PRFs* Patient Record Forms, *PHQ* Patient Health Questionnaire, *RR* response rate, *SRS* simple random sampling, *SCS* Stigma Coping Scale, *SCID-I* Structural Clinical Interview Diagnosis I, *TDM* therapeutic drug monitoring

### Publication bias

Potential publication bias was assessed by inspecting the funnel plot [71]. The funnel plots were constructed using the plot-observed studies only and plot standard error with logit event rate (see Additional file 5). In addition, statistical tests Egger’s regression test (one-tailed test), *p = 0.683*, and Begg’s rank correlation (one-tailed), *p = 0.831*, were computed to make sure that there is no evidence of publication bias on studies included in this systematic review and meta-analysis*.* In addition, the tests confirmed that there are no small-study effects in the meta-analysis.

## Results

A total of 46 studies were included in this systematic review and meta-analysis. Each study’s key findings and conclusion has summarized in detail (Table 2).

**Table 2:** The key findings and conclusions of studies included in the systematic review and meta-analysis (*n* = 46)

Author, country	Key findings (prevalence and associated factors)	Conclusion
Ibrahim et al., Nigeria [21]	MNA was 55.7%. Seeking for traditional treatment (OR, 6.5), male (OR, 3.3), low levels of insight (OR, 1.8), and low social support levels (OR, 1.5) were predictors	Psycho-education on adherence and the active involvement of the family has significant in the prevention of MNA.
Alene et al., Ethiopia [22]	The prevalence of MNA was 42.5%.	MA is low and associated with pill burden, side-effect, and exposure to social drugs.
Eticha et al., Ethiopia [23]	MNA was 26.5%. Positive attitude (AOR, 1.4), awareness of illness (AOR, 1.4), and relabel symptoms (AOR, 1.6). Khat (AOR, 0.2), illiteracy (AOR, 0.13), and older age (AOR, 0.03) were the predictors of MNA.	Schizophrenia patients were highly non-adherence to their medication. Intervention strategies focused on patient education can be helpful to improve adherence.
Kenfe et al., Ethiopia [24]	MNA was 41.2%. Forgetfulness was attributed to 78.2% of their MNA. Irregular follow-up, poor social support, and complex drug regimen were associated with MNA.	MNA among psychiatric patients in Southwest Ethiopia is high and revealed possible associated factors.
Hibdye et al., Ethiopia [25]	MNA was 51.2%. Poor social support (AOR, 5.2), stigmatized (AOR, 2.2), negative attitude (AOR, 4.6), medication frequency (AOR, 1.7), unemployment (AOR, 2.1), and Khat chewing (AOR, 2.1) were predictors.	MNA was found to be high. It has significant implications to enhance level of adherence by tackling factors through intervention program.
Anne et al., USA [26]	MNA was 28%. It was associated with perceived stigma (0.05), patient-rated severity of illness (0.05), interpersonal problems (0.02), and age 60 years or older (0.04).	Clinicians’ should give psychological support to improve adherence
Hill. et al., Ireland [27]	MNA was 24%. It was associated with less insight, negative attitudes toward medication, substance misuse, and treatment duration.	Longer treatment duration is associated with non-adherence
Moritz et al., Germany [28]	MNA was 20%. Side-effect, missing voices, feeling of power as a motive for non-compliance, stigma, mistrust against the physician, and rejection of medication were the most frequent reasons for drug discontinuation	Approximately 1-in-5 patient had discontinued antipsychotic treatment due to forgetfulness and ambivalence toward symptoms.
Mert et al., Turkey [29]	MNA for bipolar disorder, schizophrenia, and MDD was 12.1%, 18.2%, and 24.2%, respectively. Irregular follow-up (OR, 5.7) and diagnosis (OR, 1.5).	MNA is a serious problem. Ensuring regular follow-up appointments and improving their thoughts are needed.
Novick et al., Multi-country-European [30]	MA was higher in bipolar patients than in schizophrenia, which might be schizophrenic patients had lower insight than in bipolar. Better insight was associated with higher MA and had stronger therapeutic alliance, which reduce the clinical severity.	Insight and MA were found to be closely related. Insight impacts on the therapeutic alliance with mental health and associated to treatment outcomes.
Hillary, Nigeria [31]	Adherence varied from poor adherence (55.5%) through moderate (36%) to high adherence (8.5%).	More than half of the psychiatric out-patients had MNA.
Ibrahim et al., Nigeria [32]	MNA was 54.2% (schizophrenia = 62.5%, bipolar = 45.8%). Multiple dosing frequency (OR, 7.8), side-effects (OR, 6.8), cost of medications (OR, 4.1), and poly-therapy (OR, 2.3) were factors associated with MNA.	Encourage rational pharmacotherapy, consider routine lower dosing prescriptions, integrating side effects surveillance, and early intervention are recommended
Dibonaventura et al., USA [33]	MA was 42.5%. Medication side-effect and forgetfulness were 86.19% and 48.4%, respectively. Agitation (OR = 0.6), sedation/cognition (OR = 0.7), prolactin/endocrine (OR = 0.7), and side-effects (OR = 0.6) were significantly associated with MNA.	Medication side-effects and resource are associated with MNA. Prevention, early detection, and effective management of side-effects are crucial to avert it.
Gurmu, et al., Ethiopia [34]	MNA was 50.2%. Schizophrenia (75.7%), bipolar disorder (37.5%), and depression (52.6%). Factors were perceived recovery (26.7%), drug unavailability (18.1%), adverse effect (12.7%), forgetfulness (10.6%), and being busy (8.6%).	The observed rate of antipsychotic MNA in this study was high. Interventions to increase adherence are therefore crucial.
Magura et al., USA [35]	Lower social support, alcohol use, lower satisfaction with medication, side-effects, lower self-efficacy for avoidance and recovery, forgetfulness, unnatural to be controlled by medication, careless at times, and felt better were the reasons for MNA.	Health care providers should encourage to address patients’ adherence strategies via education about side-effects and benefits of the medication.
Kikkert et al., European countries [36]	Medication efficacy, external factors (such as patient support and therapeutic alliance), insight, side-effects, and attitudes had influence on MA.	Professionals, care-givers, and patients do not have a shared understanding of which factors are important.
Teferra et al., Ethiopia [37]	Inadequate availability of food, perceived strength of medications, social support and safety net, lack of insight, failure to improve, side effects, substance abuse, stigma, and poor attitude of the care provider were some of the main reasons for MNA.	Greater attention to provision of social and financial assistance will potentially improve MNA.
Sher et al., USA [38]	Caregivers’ attribution of depression to cognitive and attitudinal problems, which significantly predicted patients’ MNA. Perceived stigma was also another predictor of non-adherence.	Involving caregivers on the treatment plan, social support, and attitude may improve adherence.
Mohamed et al., USA [39]	Insight and drug attitudes were associated with declining schizophrenia symptoms but increasing levels of depression. Change toward more positive medication attitudes was associated with changes in insight, improve community functioning, and greater medication compliance.	Better insight, positive attitudes toward medication, and educational interventions can be an important part of psychosocial rehabilitation services.
Sava, Turkey [40]	MNA was 26.5% and associated with education, lack of insight, thought they had recovered, believed that treatment had no-effect on their disorder, thinking that had recovered, not taking medication, and thought of treatment not effective.	Lower education level, having thought of inadequate information about illness, and lack of insight about treatment were significantly associated with MNA.
Sirey, USA [41]	MNA was 82%. Elderly (24%) and younger (13%) patients discontinued treatment completely. Patients perceived more stigma than older patients, stigma predicted treatment discontinuation.	Patients’ perceptions of stigma at the start of treatment had influence their subsequent treatment behavior.
Sajatovic. USA [42]	MNA was 45.9%. Younger age, unmarried, homeless, substance abuse, or fewer outpatient psychiatric visits were predictors.	Almost half of the patients had MNA that reduce the effectiveness treatments in clinical settings.
Sajatovic. USA [43]	MA was 51.9%. Factors associated were younger age, comorbid substance abuse, and homelessness were the factors associated with MA level.	MNA is common in bipolar disorder medication.
John, USA [44]	MNA was 77%. Weight gain and cognitive effects of a medication most significantly affected patients’ likelihood of MA.	Patients’ satisfaction is seriously affect adherence. Health care providers can optimize prescribing patterns.
Iseselo et al., Tanzania [45]	Financial constraints, lack of social support, family disruption, stigma, discrimination, and disruptive behavior were some of the influencing factors for MNA.	A collaborative approach between the care providers, leader,and family is needed.
J.M. Olivares, Spain [46]	Minimize patients waiting stay was significantly associated with MA.	Treatment retention had greater improvement in clinical symptoms, reduce hospital stay, and increase efficacy.
Charlotte, Sweden [47]	Antidepressant MNA was 61.4%. Age (< 35 or > 64 years), having personality disorder, sensation-seeking traits, substance abuse, and unavailability of concomitant medications were predictors.	Patient and illness-related factors may imply an increased risk of MNA.
Adeponle, et al., Nigeria [48]	Half (50.6%) of patients were adherent with appointments.	Family support was significantly associated with appointment, which can improve MA.
Rashid, Malaysia [49]	The type of antidepressant medication prescribed, not given a choice to choose the treating doctor, and the preference to traditional medicine were significant risk factors.	Involvement of patients, caregivers, flexible schedule, place choice, drug, and doctor can help to prevent MNA.
Roy, Ranchi (India) [50]	Poor infrastructure and lack of proper information about mental illness to patients and caregivers were some of the reasons for MNA.	Develop community mental health care facilities and provide adequate information to patients and caregivers.
Omran, Iran [51]	Non-compliance was reported as a possible cause of admission in (88.2%) of the re-hospitalized cases. No insight to disease (59%) and feeling of cure (27.6%) were causes for MNA.	Providing a better insight about disease to patients to take their medications, even feeling of cure is important.
Tara et al., Canada [52]	MA was 73%. Forgetting, change in routine, side effect, had lower self-efficacy, female, and had not completed post-secondary education were the most frequently identified reasons for MNA.	Clinicians should be simple and easy to address medication efficacy, tolerability, and social moderator
Banerjee, India [53]	MNA was 66.9%. Women (OR 2.7), consume extra pills (OR 2.8), and had a considerably lower internal locus of control (OR 4.5) were predictors	Interventions focusing on individuals and intersectoral system-oriented approach to improve MA are needed.
Oliver, Spain [54]	MNA was 33.9%. Long-term treatment duration is a factor for MNA. Women were more adherent than men.	Designing proper drug collection at pharmacies can improve the MA of patients.
Dave, UK [55]	Discontinuation was 80%. Lower discontinuation in the first 6 months after initiation was associated with higher age, weight gain, and comorbid irritable bowel syndrome.	Lack awareness was a risk for discontinuation.
Mahaye, South Africa [56]	MNA was 50.8%. Age and race become predictors of MNA.	Age and race were significant predictors for MNA.
Sundell, Sweden [57]	MNA was 26.1%. It was less in women (OR, 0.8) and least 2 years of higher education (OR, 0.7), and those who received social assistance (OR, 1.3).	MNA occurred more commonly among social support recipient
Akincigil, [58]	MNA was 49%. Care from a psychiatrist and higher general pharmacy utilization were associated with better adherence. Younger age, substance abuse, and comorbidity were associated MNA.	Substance abuse is one of the main risk factor for MNA and needs to be targeted for intervention.
Taj, Pakistan [11]	MA among major depressive and bipolar disorders was 61.5% and 73.9%, respectively. Reasons were sedation (30%), cost (22%), forgetting (36%), and no explanation by doctors (92%).	MNA is a common and important issue. Treatment cost and co-morbidity are common factors
Prukkanone et al., Thailand [59]	MA was 41% but all patients who attended only once were non-adherent, adherence may be as low as 23%.	MA to antidepressant therapy for treatment was high.
Shigemur, Japan [60]	MNA was 33.1%. It was associated with lower age, unemployed (OR, 1.9), higher daily dosing frequency, low drug satisfaction, and poor doctor–patient dyad, and age (> 34 years) (OR, 1.6).	MNA was predicted by lower age and unemployment.
Bambouer et al., USA [61].	MNA was 75%. Rates of antidepressant non-adherence significantly increased over time were 40%.	Effectiveness of electronically triggered, patient-specific, and faxed feedback should be carefully evaluated.
Demyttenaere et al., Belgium [62]	MA was 70%, and it was decreased by 2.5% per month and more than three times more rapidly in drop-outs.	MA decreases with time is influenced by demographic and clinical variables.
Mascha C. Ten D [63].	MNA ranged from 39.7 to 52.7%. It did not significantly differ between intermittent ad continuation antidepressant users (37.2% versus 25%).	MNA is high on MDD. Doctors continuously have to be aware of this problem
Baldessarini et al., USA [64]	MNA was 33.8%. Prescribing psychiatrists considered only 6% as MNA. Alcohol, youth, comorbidity, side effects, obsessive-compulsive disorder, and recovering from mania-hypomania and drug-complexity were the predictors.	Underestimation of the problem may encourage increasingly complex treatment regimens of untested value, added expense, and risk of adverse effects
Nega et al., Ethiopia [65]	MNA was 61.2%. It was associated with female (AOR, 2.3), combined drug (AOR, 2.7), long treatment duration (AOR, 2.3), > 24 months (AOR, 2.5), substance use (AOR, 2.6), perceived stigma (AOR, 2.2), patient’s poor attitude (AOR, 3.0), and poor social support (AOR, 1.8).	Psychotropic MNA was high. We recommend the concerned bodies to design and implement programs focused on associated factors in order to improve MA.

*AOR* adjusted odds ratio, *CI* confidence interval, *MA* medication adherence, *MNA* medication non-adherence, *SCID-I* Structural Clinical Interview Diagnosis I, *TDM* therapeutic drug monitoring

### Magnitude of psychotropic medication non-adherence

**Fig. 2:**
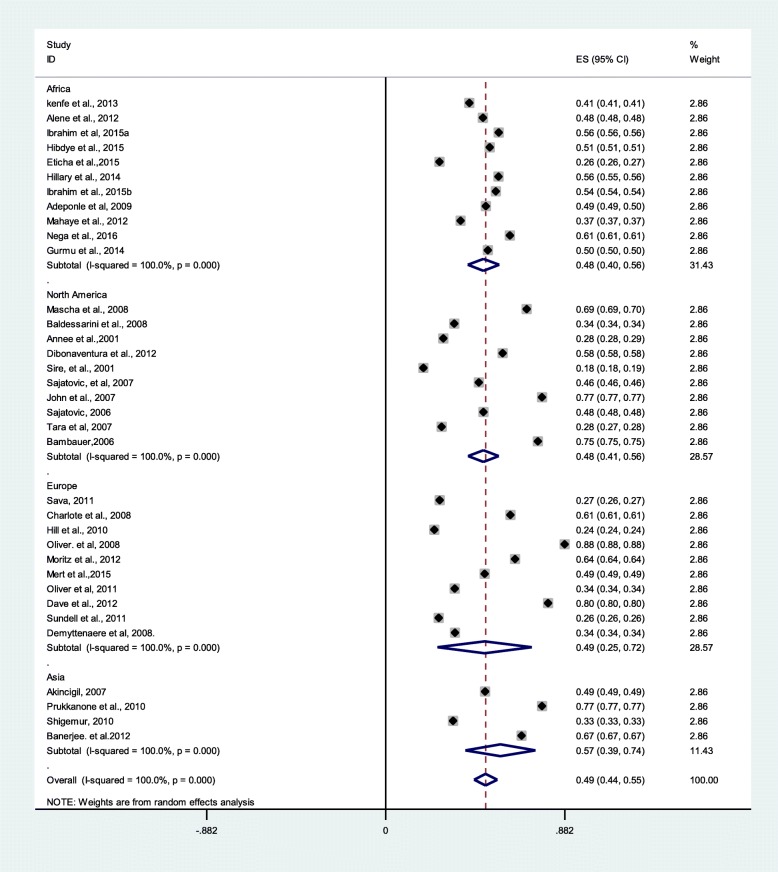
Pooled estimate of medication non-adherence (*n* = 35)

Thirty-five studies were used for meta-analysis to compute the pooled proportion of the psychotropic medication non-adherence. In 35 studies with 63,957 cases from a sample of 120,134, the pooled prevalence of medication non-adherence among major psychiatric disorders was 49% (95% CI 44%, 55%). In addition, the psychotropic medication non-adherence was 48%, 48%, 49%, and 57% in Africa, North America, Europe, and Asia, respectively (Fig. 2**)**.

### Medication non-adherence among schizophrenia patients

**Fig. 3:**
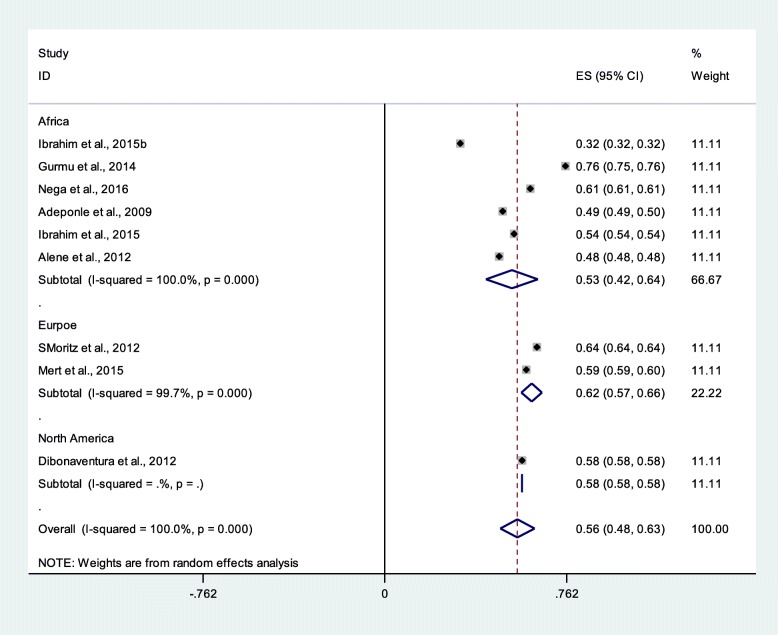
Pooled estimate of medication non-adherence of the schizophrenia patients (*n* = 9)

Sub-group analyses were conducted for studies that reported medication non-adherence among schizophrenia patients. From nine studies with 2643 participants, the medication non-adherence among schizophrenia patients was 56% (95% CI 48%, 63%). The prevalence in the sub-group analysis was relatively consistent with the overall pooled prevalence (Fig. 3).

### Major depressive disorder medication non-adherence

**Fig. 4:**
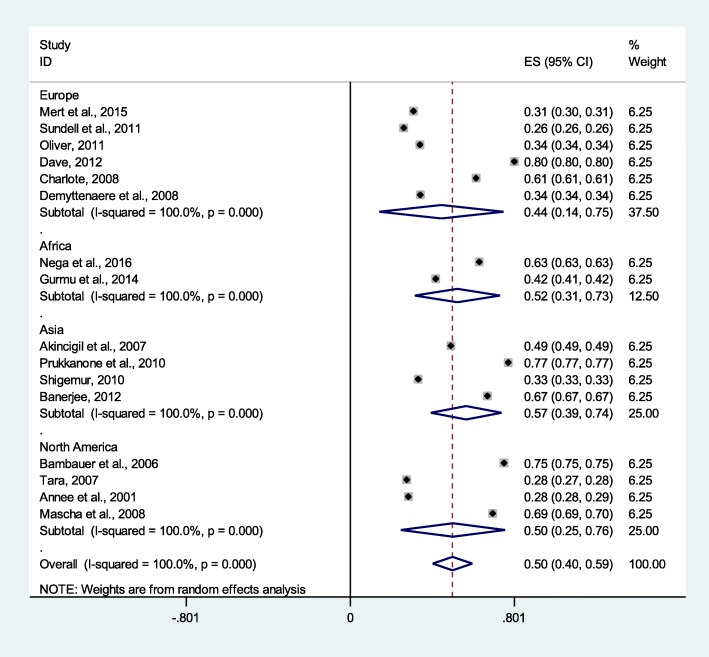
Pooled estimate of medication adherence of the major depressive disorders patients (*n* = 16)

From 16 studies with 42,255 participants, medication non-adherence among patients with major depressive disorders was 50% (95% CI 40%, 59%). The prevalence in the sub-group analysis was relatively consistent with the overall pooled prevalence, but a bit lower in Europe (Fig. 4).

### Bipolar disorder patients’ medication non-adherence

**Fig. 5:**
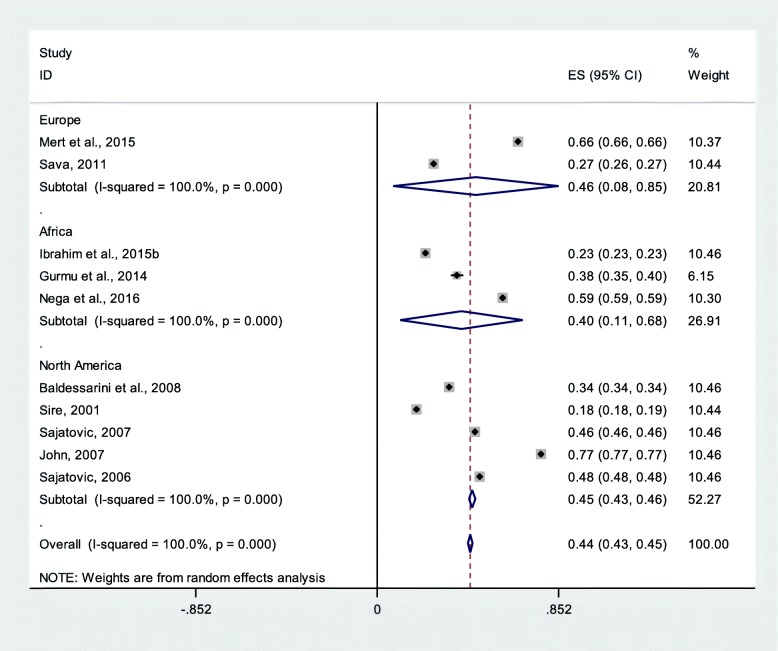
Pooled estimate of medication non-adherence of bipolar disorder patients (*n* = 10)

From 10 studies with 73,250 study participants, medication non-adherence among patients with bipolar disorders was 44% (95% CI 43%, 45%) (Fig. 5**)**.

### Determinants of psychotropic medication non-adherence

Medication non-adherence is influenced by various factors. We systematically mapped the factors that affect medication non-adherence among patients with major psychiatric disorder into individual patient, social support, clinical or treatment and illness, and health system-related factors based on the review of 46 studies.

### Factors related with individual behaviors

#### Patient’s socio-demographic factors

Some psychiatric patients’ socio-demographic characteristics were associated with medication non-adherence. However, the association was inconsistent across studies. In four studies, unemployment was one of the factors associated with medication non-adherence [25, 31, 56, 60]. On the other hand, the nature of the job (for example, engaging in farming activities, being busy) influenced patients’ adherence to their medication [50]. Educational status was one of the influencing factors of medication non-adherence. In six studies, psychiatric patients having lower education level (lower than secondary education) were more likely to be non-adherent to their psychotropic medication compared to those patients having higher educational level [23, 40, 44, 52, 56, 57]. Patients’ non-adherence to their psychotropic medication was associated with some non-modifiable demographic factors such as age and gender) [28]. In three studies, patients aged 60 years and older were more likely to be non-adherent to their medication [23, 55, 58]. Nevertheless, one study [60] reported that young age (less than 34 years) patients were also more likely to have medication non-adherence. In three studies, the relationship of gender and medication non-adherence was inconsistent. Being female was a factor associated with medication non-adherence [52, 53, 65]**,** but in two studies, being male also linked with medication non-adherence [21, 54].

### Patients’ substance abuse

In eight studies, both psychostimulant and psycho-depressant substances misuse were associated with psychotropic medication non-adherence [23, 27, 29, 37, 42, 43, 58, 65]. In three studies, psycho stimulants (e.g., cigarette smoking) was a factor associated with psychotropic medication non-adherence among major psychiatric patients [23, 25, 65]. Likewise, three studies conducted in Ethiopia [23, 25, 65] have reported that “Khat” chewing was a factor associated with psychotropic medication non-adherence among psychiatric patients. In addition, in six studies, having a history of concurrent alcohol dependency was the main factor associated with psychotropic medication non-adherence [23, 25, 37, 47, 64, 65].

### Patient attitude toward medication

In four studies, patients’ attitude toward medication was a crucial factor affecting treatment adherence and therapeutic alliance. Patients having negative attitude towards their medication was a factor associated with psychotropic medication non-adherence [23, 25, 39, 65]. Moreover, in two studies, patients having negative attitudes toward the psychotropic medication were more likely to seek alternative treatment such as traditional or religious treatment practices [21, 24]. Likewise, where patients were suspicious about the medication, believes that the medication would harm them, heard voices telling them not to take the medication, and taking medication is unnatural were less likely to adhere [36]. In three studies, psychiatric patients may also attribute to antipsychotic medication non-adherence due to the alterations in cognitive and attitudinal functioning and therefore be unwilling to use the medication [29, 37, 38].

### Patients’ perceived stigma

In eight studies, the perception or the feeling of psychiatric patients being stigmatized by their families, neighbors, health professionals, and other community members was a factor associated with medication non-adherence [24, 25, 28, 36, 37, 41, 45, 65]. In one study, both internal and external triggering factors caused the patients to feel being stigmatized. Some of these included patient believe that they can get better without medicine were afraid of medication dependency and felt too embarrassed to take the medicine [24]. On the other hand, patients perceived the effects of the medication to be unnatural and reported feeling better after terminating them were the factors associated with medication non-adherence [35]. Similarly, in two studies**,** those patients who perceived that the treatment had no effect on their illness were more likely to be non-adherent to their medication [26, 40]. In seven studies, behavioral factors such as forgetting the right dose and right time of taking medication were the factors associated with medication non-adherence [11, 22, 24, 28, 34, 35, 52]. In six studies, patients and caregivers reported being busy with daily routines, careless about the timing, forgetting to remember medication time, and irregular follow-up were associated with medication non-adherence [11, 22, 24, 34, 35, 52]. In the worst scenario, patients’ complete rejection of the medication was a main cause of discontinuation and non-adherence to their medication [28].

### Clinical factors

The clinical factors of medication non-adherence were re-categorized into medication side-effect, lack of insight about their illness and treatment, comorbidity, medication efficacy, long treatment duration, and complexity of the prescribed medication.

### Medication side-effects

In several studies, psychotropic medication non-adherence was associated with medication-related side-effects [11, 22–25, 28–30, 32–37, 39, 44, 50, 52, 55, 64, 65]. In seven studies, patients feeling dizziness, fatigue, tiredness, sedation, lethargy, and sleepiness were the most frequently reported side-effects that contributed to medication non-adherence [11, 33, 36, 37, 50, 52, 65]. In two studies, sleepiness during day time (medication dose time) and potentially life-threatening or distressing side-effects seriously affected patients’ medication non-adherence [37, 44]. Another two studies, feeling of powerlessness, insomnia, difficulty thinking or concentrating, restlessness, or feeling jittery were found to be associated with medication non-adherence [28, 37]. Likewise, in five studies, weight gain was another medication-related side-effect that associated with medication non-adherence and patients’ perception toward their medication [25, 33, 36, 44, 55].

In two studies, side-effects such as decreased sexual interest and having a symptom of sexual dysfunction were associated with patients’ medication adherence [33, 36]. Moreover, patients and caregivers’ perceived medication adverse drug reaction was a factor associated with psychotropic medication non-adherence [34, 36]. Likewise, extra pyramidal symptoms or agitation [33], other medication-related side-effects such as cognitive deterioration or impairment [44], missing voice [28], paralysis of body parts, twisting of the neck, drooling, weakness, appetite stimulation [37], severe depressive symptoms and episodes [39, 44], salivation, dry mouth, and memory problem [50] were common factors associated with medication non-adherence.

### Lack of insight about illness and medication

In seven studies, patients’ lack of insight (level of awareness or understanding) about their illness and medication was a common factor associated with psychotropic medication non-adherence [21, 23, 28, 39, 40, 50, 51]. Likewise, misunderstanding about the treatment consequences, lack of awareness of their illness and or mental disorder in general, and sometimes appreciating subjective relief symptoms [30, 36, 37] were the factors associated with medication non-adherence among major psychiatric disorder patients.

### Medication efficacy

The pharmacological management of psychiatric disorders needs safe and efficacious medication to achieve desired treatment goals. The fact that lower medication efficacy and patient self-rating of efficacy were also factors associated with psychotropic medication non-adherence. Taking lower potent concomitant psychotropic medications [47, 65], recovery from illness [34], felt better [24, 52], and failure to improve with medication [37] were the factors associated with medication efficacy related with psychotropic medication non-adherence. Likewise, patients’ or caregivers’ perceived medication efficacy such as subjective relief of symptoms, patients’ feel drugs have no effect on the illness, not helpful, being ineffective [36, 46, 50], and feeling of cured [51] were side-effect-related factors associated with medication non-adherence.

### Medication duration

In five studies, long treatment duration (6–12 months and longer) was an associated factor for medication non-adherence [22, 25, 37, 43, 65]. Similarly, having long-term medication prescriptions, long duration maintenance therapy [29, 54], and irregular follow-up [29] were associated with psychotropic medication non-adherence.

### Treatment complexity

In three studies, multiple dose, frequency and drug combinations, or complex drug regimen were seriously linked with medication non-adherence [24, 32, 64]. In two studies, pill burden or consuming extra pills was also one of treatment-related factors that negatively influenced patients’ adherence to their psychotropic medication [22, 53]. In another two studies, taking medication twice per day was a negative factor for medication adherence [46, 49]. In addition, the route of medication administration had a significant effect on medication non-adherence. The patients on injectable medication were more likely to be adherent than the patient taking drugs orally [46].

### Co-morbidity

In three studies, psychotropic medication adherence was compromised where there were co-morbidities of mental illness and other physical illnesses. Studies [11, 43, 64] reported that medication non-adherence was associated with patients having co-morbidities with their current psychiatric disorders. Of these, affective morbidity, obsessive-compulsive disorders, recovering from mania-hypomania [64], personality disorders and sensation of seeking personality traits [47], and alcohol abuse disorders [61] were negatively associated with medication adherence. Irritable bowel syndrome as a co-morbidity was also significantly associated with medication non-adherence [55].

### Lack of social support

In seven studies, poor or lack of social or family support was associated with psychotropic medication non-adherence [21, 24, 25, 35, 37, 45, 65]. In two studies, limited or inadequate patient information, weak professional or family support, therapeutic alliance, social involvement, and low education were some of the social support-related factors [36, 37]. Cohesiveness, family reminding, and transport to hospital [37], lower family harmony or lack of resilient family support, discrimination by nearby people, disruption of family functioning or household routine and religious practices [45], weak community functioning [39], homelessness [42, 43], had old age caregivers or lack of caregivers [50], lack of family compliance of follow-up [51], lack of advice about their medication intake from friends and relatives [28], not receiving social assistance [57], and caregivers’ attribution of depression to cognitive and attitudinal problem [38] were the factors associated with psychotropic medication non-adherence.

### Health system-related factors

The health system-related factor was the crucial area for getting quality mental health service. In three studies, medication non-adherence was associated with lack of free access to medicine due to inadequate or unavailability of psychotropic drug supplies in health facilities [24, 25, 34]. In one study, although psychotropic medications were normally provided free of charge in the government health facilities, patients were suffering unavailable of medication in the government pharmacies. Thus, patients need to buy from private pharmacies which are very expensive and lead to interruption of the medication. In addition, health care provider sometimes changes the drug but it may not be found in the government hospital pharmacy [45]. Therefore, the lack of alternative drug or therapy affects psychotropic medication adherence [39]. On the other hand, the lack of sufficient and quality health education to psychiatric patients and or their caregivers/relatives/families about the medication and illness influenced patients’ adherence to their prescribed medication [22, 36, 39, 40, 51].

In three studies, patient-physician or therapist relationship was crucial for better medication adherence. Consequently, unfriendly, judgmental behavior, inflexible appointment systems, mistrust, and having negative patient-physician relationships were the factors associated with patients’ psychotropic mediation non-adherence [28, 30, 60]. In one study, health care providers’ negative attitude had influenced patients’ adherence to the medication and their follow-ups [37]. Similarly, in two studies, health professional shortages had also affected medication adherence [49, 58]. Patient preference for traditional/complementary medicine was another cause of medication non-adherence [49]. Medication non-adherence was affected by the number of hospitalizations [28], irregular hospitalization and frequently discharge of patients, length of stay [42, 46], lack of patients’ satisfaction with health care services [44], and long distance to access the health service/recollect medications [50]. In three studies, health care providers would be unable to explain and optimize prescribing pattern, timing, and dose benefit of medication. In addition, the lack of friendly deal with medication complexity, tolerability, efficacy, and health belief issues were critical factors influence medication adherence [11, 44, 52]. Furthermore, the health care system has also associated with medication non-adherence. These factors were poor service structure and cumbersome purchasing procedure (affect access), availability and timely use or collection of psychotropic medication during follow-up visit, and patients not covered by health insurance scheme [58].

### Medication cost

In seven studies, psychiatric patients and their caregivers having financial constraints to buy medicines were factors associated with medication non-adherence. In addition, the lack of money for transportation, to purchase proper food, and to buy medications were the factors associated with patients’ adherence to their medication. Psychotropic medications had an appetite stimulation that has been increasing food demand which incurs an additional economic burden [11, 24, 32, 37, 42, 45, 50] and contribute for medication non-adherence.

## Discussion

This systematic review and meta-analysis determined the pooled proportion of psychotropic medication non-adherence and synthesized the associated factors with medication non-adherence among major psychiatric disorder patients. Almost half (49%) of patients with major psychiatric disorders did not adhere to their psychotropic medication. Medication non-adherence among patients with schizophrenia, major depressive disorder, and bipolar disorder were 56%, 50%, and 44%, respectively. Medication non-adherence is influenced by various factors such as patients’ individual behavior, social or family support, clinical or illness and treatment-related, and overall health care system-related factors.

Previous systematic reviews have indicated that medication non-adherence is a common challenge in the treatment of psychiatric disorders [72, 73]. This meta-analysis finding is consistent with a systematic review revealed an overall medication adherence level of 58% (ranged from 24 to 90%), and medication adherence to antidepressants was 65% [6]. Another earlier systematic review has shown that the level of medication non-adherence was 60% [74]. The present systematic review and meta-analysis finding is consistent with a finding from a comprehensive systematic review on schizophrenia which reported that a mean rate of non-adherence was 41.2%. The sub-group analysis indicated that a mean non-adherence rate was 49.5% [14], and another systematic review has shown that psychotropic mediation non-adherence was 44% [75]. Nevertheless, the present meta-analysis finding is a bit lower than a finding from a systematic review which revealed that adherence in psychiatric patients ranged from 10.7 to 38% [76].

This systematic review of factors influencing psychotropic medication non-adherence which is consistent with other systematic reviews [14, 72, 73, 77] has shown that medication adherence is mainly affected by patients’ negative attitude toward their medication, lack of insight, negative health belief, and perceived stigma. Similarly, medication non-adherence is consistently associated with patients behavioral practices (e.g., substance abuse) [14, 74] and also patients’ socio-demographic characteristics (such as educational status, age, gender, and employment) [14, 72]. The present systematic review has identified that the lack of social support is associated with medication non-adherence among major psychiatric disorder patients. This is similar with other reviews, which have reported that the lack of family involvement, care/dyad support, and other social supports are strongly negatively associated with poorer therapeutic alliance [14, 72, 75, 76]. In addition, medication non-adherence is associated with clinical- or medication-related factors [14, 72–74, 77]. This finding is supported by another systematic review which revealed that psychiatric disorder comorbidities with other physical disorders influence medication adherence and increase re-admission of psychiatric patients [73, 78, 79].

In the present systematic review, medication non-adherence is associated with poor functioning of the health system such as lack of psychotherapy, lack of information, long treatment duration with little health personnel follow-up, inadequate discharge planning, increased hospitalizations, poor support and care environment, experiencing access barriers to high-quality care and health care providers unable to provide elicit information on adherence, inadequate medication coverage, and poorer therapeutic alliance [14, 72, 74–76, 79]. Financial factors seriously affected medication adherence. These included unaffordability of medication, increased health care cost [73, 74], lack of health insurance [75], patients’ poor capacity, and limited resources [73].

A large amount of heterogeneity in the definition and measurement methods used to assess medication adherence have been reported in some reviews. The heterogeneity of factors related to non-adherence calls for individually tailored approaches to promote adherence [80, 81]. Non-adherence contributes enormously to poor health outcome and needs substantial work to improve treatment outcomes [80]. Evidence showed that improving adherence to psychotropic medications could have a positive impact on patients and society. Non-adherence issues need to be looked at from many angles and taking a multifaceted approaches with patients and healthcare providers to address identified challenges [81].

## Conclusions

Almost half of patients with major psychiatric disorder did not adhere to their psychotropic medication. Patients’ individual behavior, lack or poor social/family support, treatment and illness-related clinical conditions, and the health system barriers are influencing factors of psychotropic medication non-adherence among patients with major psychiatric disorders. Therefore, multifaceted intervention is needed to create supportive environment for patients and caregivers to minimize psychotropic medication non-adherence. Additionally, supportive social and health care system programs should be designed to alleviate major psychiatric disorder patients’ medication non-adherence. Comprehensive approaches targeting the factors that affect medication non-adherence can bring tremendous positive outcomes. This systematic review and meta-analysis finding can be helpful to inform policy-makers, clinicians, and other caregivers to undertake necessary decisions to establish an integrated approach to boost therapeutic alliance and improve medication adherence.

## Supplementary information

**Additional file 1.** PRISMA checklist

**Additional file 2.** Sample searching strategies

**Additional file 3.** Newcastle Ottawa Scale (NOS)

**Additional file 4.** Data extraction on Excel sheet

**Additional file 5. **Funnel plot for exploration of publication bias (*for overall pooled and subgroup analysis*)

## Data Availability

The data that support the review findings are available upon submitting a reasonable request to the corresponding author.

## Abbreviations

AOR: Adjusted odds ratio
CI: Confidence interval
MA: Medication adherence
MNA: Medication non-adherence
NRR: No response rate
PRISMA: Preferred Reporting Items for Systematic Review and Meta-Analysis
PROSPERO: Prospective Register of Systematic Reviews
RR: Response rate
TDR: Tropical Disease Research
US: United States
WHO: World Health Organization
