# Supplementary material for: Psychotropic medication non-adherence and its associated factors among patients with major psychiatric disorders: a systematic review and meta-analysis
Source: Syst Rev. 2020 Jan 16;9:17. doi: 10.1186/s13643-020-1274-3 (PMC6966860; doi:10.1186/s13643-020-1274-3)
Supplement: Supplementary file 3 — Additional file 3. Newcastle Ottawa Scale (NOS) [file 13643_2020_1274_MOESM3_ESM.docx]

**Newcastle - Ottawa Quality Assessment Scale** for observational studies

*Note: each item account 1 point. (Accept the study if total score ≥7)*

| **First author, publication year** | **Criteria** | | | | | | | | |  |  |  |  |
| --- | --- | --- | --- | --- | --- | --- | --- | --- | --- | --- | --- | --- | --- |
|  | **Selection** | | | | **Comparability** | | **Exposure** | | |  |  |  |  |
|  | **Is the case definition adequate** | **Representativeness of the cases** | **Selection of Controls** | **Definition of Controls** | **study controls for most important factor** | **study controls for second important factor** | **Ascertainment of exposure** | **Same method of ascertainment for cases and controls** | **Non-RR** | **Event rate (n/N)** | **Effect size (proportion)** | **Quality score** | **Decision** |
| Ibrahim et al, 2015. (CS) | **1** | **1** | **1** | **1** | **1** | **UC** | **1** | **1** | **1** | **206/370** | **0.55** | **8** | **Accept** |
| Alene et al., 2012, (CS) | **1** | **0** | **1** | **1** | **1** | **UC** | **1** | **1** | **1** | **161/336** | **0.48** | **7** | **Accept** |
| Eticha,T. et al, 2015 (CS) | **1** | **1** | **1** | **UC** | **1** | **UC** | **1** | **1** | **1** | **104/393** | **0.26** | **7** | **Accept** |
| Kenfe T. et al, 2013. (CS) | **1** | **1** | **1** | **UC** | **1** | **UC** | **1** | **1** | **1** | **174/422** | **0.41** | **7** | **Accept** |
| Hibdye et al. 2015, (CS) | **1** | **1** | **1** | **UC** | **1** | **UC** | **1** | **1** | **1** | **210/410** | **0.51** | **7** | **Accept** |
| Joo Anne et al. 2001 ( Longitudinal) | **1** | **1** | **1** | **1** | **1** | **UC** | **1** | **1** | **1** | **38/134** | **0.28** | **8** | **Accept** |
| Michael Hill. et al, 2010. (pros. CH) | **1** | **0** | **1** | **1** | **1** | **UC** | **1** | **1** | **0** | **41/171** | **0.24** | **7** | **Accept** |
| S. Moritz et al, 2012 (Pros. CH) | **1** | **0** | **1** | **1** | **1** | **UC** | **1** | **1** | **0** | **72/1130** | **0.64** | **7** | **Accept** |
| Mert D. G. et al. 2015. (CS) | **1** | **1** | **0** | **1** | **1** | **UC** | **1** | **1** | **0** | 99/203 | 0.49 | **6** | **Accept** |
| Novick et al. 2015 (Pros. CH) | **1** | **1** | **1** | **1** | **1** | **UC** | **1** | **1** | **0** | SR | SR | **7** | **Accept** |
| Hillary O. Odo,2014 (CS) | **1** | **0** | **1** | **1** | **1** | **UC** | **1** | **1** | **1** | 111/200 | 0.56 | **7** | **Accept** |
| Ibrahim et al,2015 (CS) | **1** | **1** | **1** | **1** | **1** | **UC** | **1** | **1** | **1** | 194//358 | 0.54 | **8** | **Accept** |
| Dibonaventura et al. 2012 (CS) | **1** | **1** | **0** | **1** | **1** | **UC** | **1** | **1** | **1** | 504/876 | 0.58 | **7** | **Accept** |
| Gurmu, et al. 2014. (CS) | **1** | **1** | **1** | **1** | **1** | **UC** | **1** | **1** | **1** | 105/209 | 0.50 | **8** | **Accept** |
| Magura et al. 2012(CS) | **1** | **1** | **1** | **1** | **1** | **UC** | **1** | **1** | **0** | SR | SR | **7** | **Accept** |
| M. J. Kikkert et al. 2006 (Qualitative) | **1** | **0** | **0** | **UC** | **0** | **UC** | **1** | **1** | **0** | SR | SR |  | **Accept** |
| Teferra et al,2013 (qualitative) | **1** | **0** | **1** | **UC** | **0** | **UC** | **1** | **1** | **0** | SR | SR |  | **Accept** |
| Iris Sher et al, 2005.(longitudinal) | **1** | **1** | **1** | **1** | **1** | **UC** | **1** | **1** | **1** | SR | SR | **8** | **Accept** |
| S. Mohamed et al. 2008. (baseline trial) | **1** | **1** | **1** | **1** | **1** | **UC** | **1** | **1** | **1** | SR | SR | **8** | **Accept** |
| Haluk A. Sava, 2011. (CS) | **1** | **1** | **0** | **1** | **1** | **UC** | **1** | **1** | **1** | 39/147 | 0.27 | **7** | **Accept** |
| Jo Anne Sirey, 2001. (CS) | **1** | **1** | **0** | **1** | **1** | **UC** | **1** | **1** | **1** | 17/92 | 0.18 | **7** | **Accept** |
| Martha Sajatovic. 2007. (Longitudinal | **1** | **1** | **1** | **1** | **1** | **UC** | **1** | **1** | **0** | 20488/44637 | 0.49 | **7** | **Accept** |
| Martha Sajatovic. 2006. (Longitudinal) | **1** | **1** | **1** | **1** | **1** | **UC** | **1** | **1** | **0** | 12980/26986 | 0.48 | **7** | **Accept** |
| F. Reed John, 2007. USA (CS) | **1** | **1** | **1** | **UC** | **1** | **UC** | **1** | **1** | **1** | 361/469 | 0.77 | **7** | **Accept** |
| Iseselo et al, 2016 (Qualitative) | **1** | **0** | **1** | **UC** | **0** | **UC** | **1** | **1** | **0** | SR | SR |  | **Accept** |
| J.M. Olivares, 2008. (Pros CH) | **1** | **1** | **1** | **1** | **1** | **1** | **1** | **1** | **1** | 441/550 | 0.88 | **8** | **Accept** |
| Anne Charlotte, 2008. (CS) | **1** | **1** | **1** | **1** | **1** | **UC** | **1** | **1** | **0** | 633/1031 | 0.61 | **7** | **Accept** |
| Adeponle, et al, 2009(Pros CH) | **1** | **0** | **1** | **1** | **1** | **UC** | **1** | **1** | **1** | 40/81 | 0.49 | **7** | **Accept** |
| Ak Rashid,2010 (CC) | **1** | **1** | **1** | **1** | **0** | **UC** | **1** | **1** | **1** | SR | SR | **7** | **Accept** |
| Rakha Roy 2005, (CS) | **1** | **1** | **1** | **1** | **1** | **UC** | **1** | **1** | **0** | SR | SR | **7** | **Accept** |
| Victoria Omran, 2008. (CS) | **1** | **1** | **1** | **1** | **0** | **UC** | **1** | **1** | **1** | SR | SR | **7** | **Accept** |
| Tara A, et al. 2007. (Dta RCT) | **1** | **1** | **1** | **1** | **1** | **UC** | **1** | **1** | **0** | 22/80 | 0.28 | **7** | **Accept** |
| Banerjee S, 2012, (CS) | **1** | **1** | **1** | **UC** | **1** | **UC** | **1** | **1** | **1** | 160/239 | 0.34 | **7** | **Accept** |
| Silvia Oliver, 2011.(CS) | **1** | **1** | **1** | **UC** | **1** | **UC** | **1** | **1** | **0** | 72/212 | 0.34 | **7** | **Accept** |
| Shreya Dave, 2012. ()Ret CH | **1** | **1** | **1** | **1** | **1** | **UC** | **1** | **1** | **1** | 11149/13927 | 0.80 | **7** | **Accept** |
| Sanele Mahaye, 2012 (CS) | **1** | **1** | **1** | **1** | **1** | **UC** | **1** | **1** | **0** | 35/95 | 0.37 | **7** | **Accept** |
| Karolina Andersson Sundell,2011,(CS) | **1** | **1** | **1** | **1** | **1** | **UC** | **1** | **1** | **0** | 1706/6536 | 0.26 | **7** | **Accept** |
| Ayse Akincigil, 2007 (Ret CH) | **1** | **0** | **1** | **1** | **1** | **UC** | **1** | **1** | **1** | 2113/4312 | 0.49 | **7** | **Accept** |
| Fawad Taj, 2008. (CS) | **1** | **1** | **1** | **1** | **1** | **UC** | **1** | **1** | **1** | SR | SR | **8** | **Accept** |
| Prukkanone et al, 2010. (Retr CH) | **1** | **1** | **1** | **1** | **1** | **UC** | **1** | **1** | **0** | 815/1058 | 0.77 | **7** | **Accept** |
| Jun Shigemur, 2010. (CS) | **1** | **0** | **1** | **1** | **1** | **UC** | **1** | **1** | **1** | 381/1151 | 0.33 | **7** | **Accept** |
| Bambouer K.Z et al, 2006. (CH) | **1** | **1** | **1** | **UC** | **1** | **UC** | **1** | **1** | **1** | 9846/13,128 | 0.75 | **7** | **Accept** |
| Demyttenaere k et al, 2008. (Data RCT) | **1** | **1** | **1** | **1** | **1** | **UC** | **1** | **1** | **0** | 29/85 | 0.34 | **7** | **Accept** |
| Mascha C. Ten D. 2008 (Pros CH) | **1** | **1** | **1** | **1** | **1** | **UC** | **1** | **1** | **1** | 91/131 | 0.69 | **8** | **Accept** |
| Baldessarini R.J, et al, 2008. (CS) | **1** | **1** | **1** | **UC** | **1** | **UC** | **1** | **1** | **1** | 145/429 | 0.34 | **7** | **Accept** |
| Nega M. et al, 2016, (CS) | **1** | **1** | **1** | **UC** | **1** | **UC** | **1** | **1** | **1** | 375/613 | 0.61 | **7** | **Accept** |

Newcastle-Ottawa Quality Assessment Scale for observational studies

• Selection (4)

• Comparability (1)

• Exposure (3)

NB: A study can be awarded a maximum of one star for each numbered item within the Selection and Exposure categories. A maximum of two stars can be given for **Comparability**

Note That 1=Yes, 0=No, UC=unclear
