# Supplementary material for: Psychotropic medication non-adherence and its associated factors among patients with major psychiatric disorders: a systematic review and meta-analysis
Source: Syst Rev. 2020 Jan 16;9:17. doi: 10.1186/s13643-020-1274-3 (PMC6966860; doi:10.1186/s13643-020-1274-3)
Supplement: Supplementary file 5 — Additional file 5. Funnel plot for exploration of publication bias (for overall pooled and subgroup analysis) [file 13643_2020_1274_MOESM5_ESM.docx]

Funnel plot for **overall studies** included in the meta-analysis (35 studies)

Funnel plot **schizophrenia** patients medication non-adherence (9 studies)

Funnel plot for **major depressive disorders** patients’ medication non-adherence (16 studies)

Funnel plot for **bipolar disorder** medication non-adherence studies (10 studies)
